# To Our Wives and Sweethearts—A Toast

**Published:** 1910-07

**Authors:** 


For Texas Medical Journal.

### To Our Wives and Sweethearts—A Toast.

At a banquet by the Bell County Medical Society recently Dr. W. L. Crosthwait, of Holland, was called on to respond to the toast, "To Our Wives and Sweethearts." He did it in the following eloquent and pleasing style. The "Red Back" secured a copy, and is much pleased to herewith present it to its readers.

Dr. Crosthwait said:

*Mr. Toastmaster and Gentlemen:*

In mythology we read of Aesculapius, the God of Medicine, and of Hygiae, his beautiful daughter, who was called the Goddess of Health, and in history we read of Hypocrates and of his great works and that he was called the Father of Medicine. And in later times we read of Galan and later of Genner and of Harvey, but nowhere do we find the slightest insinuation or reference that either of them ever had a wife. But let it be said of those truly great physicians of the recent past that the autobiographies of none of them have been thought complete which did not pay a just and loving tribute to the fair and faithful queen of their hearthstone.

Then I am truly glad that to me was given a theme so full of sentiment and so rich in inspiration. I am truly glad to have the honor to respond to the toast, "The Doctors' Wives"—to our wives! To them the uncrowned queens of our homes; to them who to us are the sweetest smiles of heaven. To those dear ones who with the shuttle of love have woven into the dull and lusterless warp of our lives the silver threads of happiness and the golden strands of contentment. To those who have entwined about our hearts the sweetest violets of affection and have garlanded our unworthy brows with the most fragrant roses of love.

In all ages of the world woman has been the real source of all that is good, pure, heroic and noble in the life of man. There is not a one of us around this festive board tonight who cherishes an honorable ambition or who seeks the accomplishment of a worthy achievement who did not draw his inspiration from his hearthstone. It is from the fireside where the faithful wife awaits our coming when the day's work has ended or for our return from the long, dark midnight journey that comes the courage with which we fight the battle of life and the hope that inspires us to greater efforts.

The world laughs when we laugh, but weeps not when we weep. When success crowns our efforts, the world applauds and cheers us on but leaves us deserted to silently meditate on the hour of failure. Then happy must be the doctor who labors not to gain the plaudits of the world but who works to gain the praise and approval of the wife at home, for, after all, it is she who, alone, shares with him all of the triumphs and defeats of life and all of his joys and sorrows and laughter and tears.

If we have sacrificed much in pursuing a life devoted to the cause of humanity, she has sacrificed more by sharing it with us. Magnificent colleges may perpetuate the great and good works of the truly great physician. We may read upon stately blocks of marble of his noble deeds and of his devotion to the cause of science and humanity. His name may be written in letters of gold high in the temple of fame, but where shall the memory of the noble lives and devotion to the true physicians' wives be cherished and preserved to latest posterity except in the hearts of true and perfected manhood?

The purity, devotion, charity and virtues of the highest types of womanhood typical of the true physician's wife might well be the chiefest inspiration for the poet, or might well be the most perfect model for the sculptor or the most beautiful subject for the painter, or it might well be the most eloquent theme for the orator. But such loveliness of character and such unequaled fidelity could not be sung by the poet, could not be fashioned on inanimate marble by the hand of the sculptor or portrayed upon canvas by the greatest painter, neither could it be told in the most eloquent and burning words of the world's greatest orator.

So, physician friends, let us with most sacred reflections drink to the health and happiness of our wives and wives-to-be. Let each man drink to the woman who loves him.

Then here's to our wives and sweethearts;
May our sweethearts soon become our wives,
And may our wives always be *our sweethearts.*

